# Resuscitation during robotic-assisted pelvic surgery: impact of simulation training and system-specific factors

**DOI:** 10.1007/s11701-026-03399-1

**Published:** 2026-04-13

**Authors:** Anke Hübler, Fabian Hensel, Felix Niebhagen, Roman Herout, Sherif Mehralivand, Robert Benedikt Hoeh, Anne Weber, Thea Koch, Christian Thomas, Martin Baunacke

**Affiliations:** 1 Department of Anaesthesiology and Intensive Care Medicine, Medical Faculty Carl Gustav Carus, TU Dresden, Dresden, Germany; 2 Department of Urology, Medical Faculty Carl Gustav Carus, TU Dresden, Fetscherstr. 74, 01307 Dresden, Germany

**Keywords:** Robotic-assisted surgery, CPR, Resuscitation, Simulation, Emergency management

## Abstract

Intraoperative cardiac arrest during robotic-assisted surgery is rare but poses challenges due to limited patient access and the need for undocking. Evidence on structured team training and system-specific factors affecting time to cardiopulmonary resuscitation (CPR) remains limited. This prospective single-center simulation study evaluated six multidisciplinary teams managing standardized cardiac arrest scenarios with the daVinci^®^ and HugoRAS^®^ system. Teams completed three simulations: an unannounced baseline scenario, a second scenario following structured debriefing and introduction of a standardized emergency flowchart, and a third scenario after 3–5 months to assess skill retention. Primary endpoint was time to CPR initiation. Secondary endpoints were protocol adherence and undocking time. Repeated training significantly reduced time to CPR (58.2 ± 26.2 s vs. 31.8 ± 8.9 s; *p* = 0.03) and improved compliance with the emergency flowchart (*p* = 0.03). While overall time to CPR initiation did not differ between robotic systems (daVinci^®^: 48.0 ± 23.5 vs. HugoRAS^®^: 43.2 ± 16.1, *p* = 0.6), undocking time was significantly shorter with the daVinci^®^ compared to the HugoRAS^®^ system (20.8 ± 2.4 s vs. 41.0 ± 7.5 s; *p* < 0.001). Undocking phase accounted for 48.9 ± 14.0% of the time leading up to the CPR in DaVinci^®^ and 69.8 ± 13.1% in HugoRAS^®^. Simulation-based multidisciplinary team training may improve resuscitation performance during robotic-assisted surgery. Undocking was the main source of delay with system-specific differences. Standardized emergency protocols and regular simulation training should be implemented to enhance patient safety in robotic operating rooms.

**Supplementary Information:**

The online version contains supplementary material available at 10.1007/s11701-026-03399-1.

## Introduction

Robotic surgery is becoming increasingly common in Western countries over the past two decades and is now considered standard of care for many procedures, particular in urology [1, 2]. Whereas robotic procedures were initially performed predominantly in younger and healthier patients, advances in perioperative management and surgical techniques have broadened the indications [3]. As a result, older patients and those with multiple comorbidities are increasingly undergoing robot-assisted surgery, often as an alternative to open approaches [4–6]. In addition, there is a trend, particularly in urology, towards patients undergoing surgery becoming increasingly older [6, 7]. With an older and a more comorbid population, the question arises as to whether there is a higher risk of intraoperative emergencies requiring resuscitation and how to deal with this when a complex robotic system is connected to the patient.

Restricted access to the patient and the need for rapid undocking of the robotic system may delay the initiation of life-saving measures such as chest compression and defibrillation. Large cohort studies conducted from the onset of an emergency to the start of cardiopulmonary resuscitation (CPR) show that just a few minutes can have a significant impact on the survival rate of those affected [8, 9]. For example, the survival rate improves significantly from 14.7% to 17.1% if CPR is started in less than 2 min [10]. Thus, the undocking time could have a relevant influence.

Despite these challenges, evidence on the management of intraoperative cardiac arrest during robotic-assisted surgery remains scarce. The existing literature is limited to a small number of case reports, providing little guidance on optimal team coordination, emergency algorithms, or system related differences [11–15].

At our institution, a high volume of robotic assisted procedures across multiple surgical specialties has highlighted the need for structured preparation of rare but potentially catastrophic intraoperative emergencies. To address this issue, we have developed an emergency guide and trained our local staff introducing the flowchart as part of the training. The aim of this study was to evaluate the impact of structured simulation-based training on resuscitation performance during simulated robotic-assisted surgery and to assess whether different robotic systems influence key time intervals during emergency management.

### Materials and methods

### Simulation setting and scenario

In this prospective single-centre study, six teams conducted simulated resuscitation training as part of a robotic surgery simulation between September 2024 to September 2025. Three teams performed this on a daVinci Xi^®^ system (Intuitive Surgical, Sunnyvale, USA) and three teams on a HugoRAS^®^ system (Medtronic, Dublin, Irleand). Each team consisted of a surgeon (senior physician), a surgical assistant, a sterile surgical nurse, a non-sterile surgical nurse, an anaesthetist and an anaesthesia nurse. All participants had at least one year of professional experience with the respective robotic system. The teams were not fixed across the three rounds. The teams were determined based on the members’ familiarity with the robotics system and the number of rounds they had already completed. As part of the hospital’s mandatory annual CPR training, all participants are familiar with simulated CPR procedures and know how to perform CPR.

These were in-situ simulations, meaning they took place in the regular operating rooms. Staff and resources were allocated specifically for the simulations to ensure that regular surgical operations were not disrupted [16]. In the operating theatre, the resuscitation manikin Laerdal Little Anne Simulator (Laerdal Medical, Stavanger, Norway) was combined with a conventional laparoscopic pelvic trainer.

**Fig. 1:**
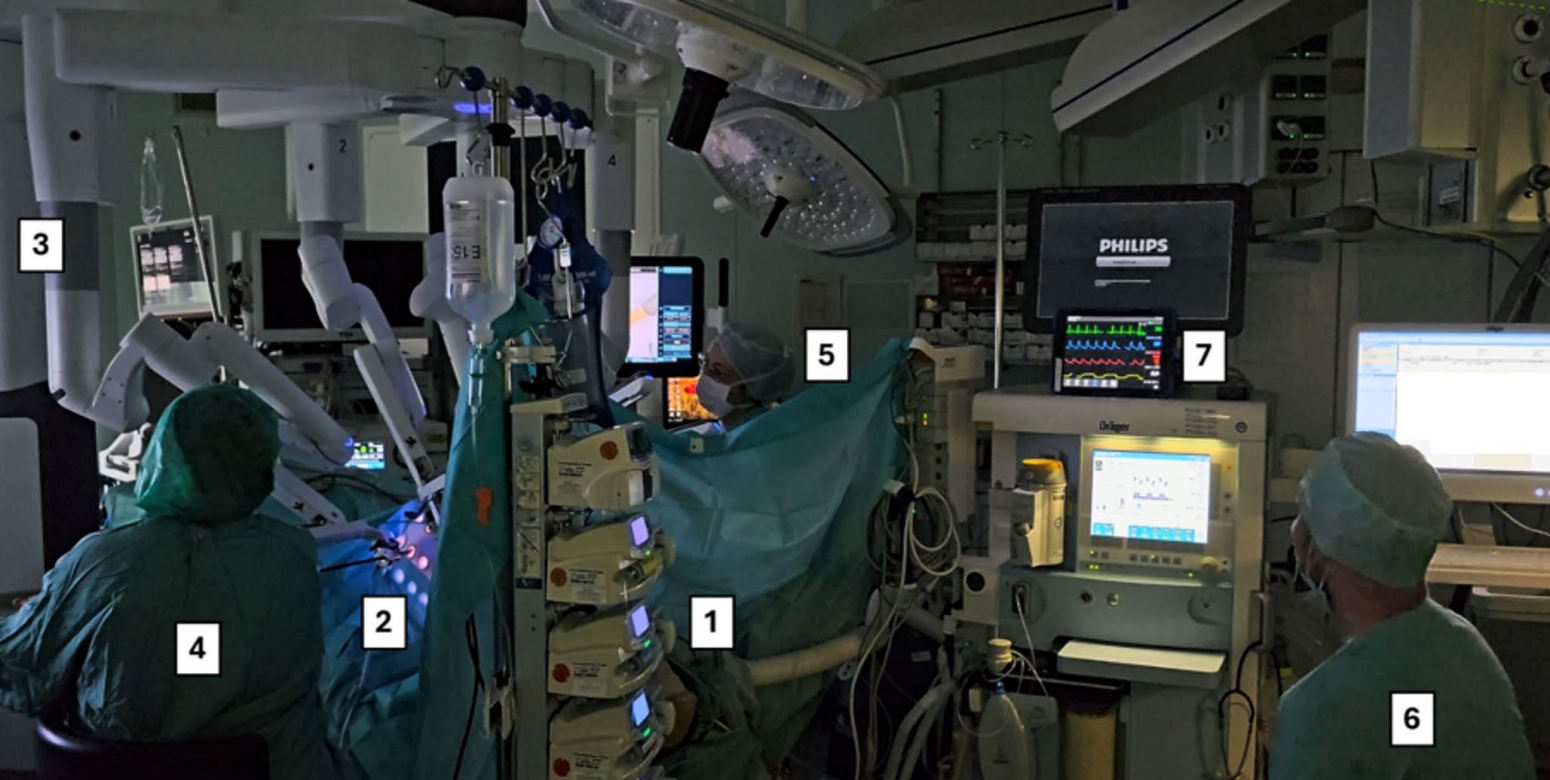
Example setting of an operating room during the simulation: The room is darkened. The resuscitation dummy is completely covered with surgical drapes [1]. The dummy’s abdomen forms the laparoscopy trainer [2]. The robotic system is docked for a pelvic procedure in the Trendelenburg position [3]. The sterile nurse sits on the left [4], the bedside assistant on the right [5]. At the head of the bed is the anaesthetist [6] with the simulation system monitor [7]

The robotic systems were connected to the LAP Trainer via four trocars, with three instruments and one camera inserted. The operating table was in a 45° Trendelenburg tilt, reflecting the standard setup for the majority of urological pelvic operations. The Little Anne simulator was intubated and ventilated. Standard intraoperative monitoring was applied, including a 5-lead electrocardiogram, pulse oximetry, invasive arterial blood pressure monitoring, one three-lumen central venous line, one peripheral venous line and a gastric tube. The mannequin was covered with sterile drapes to replicate the intraoperative environment (Fig. 1). The simulation scenario began with a brief standardized introduction. All trocars were placed, and carbon dioxide insufflation was called initiated. After positioning, operating room lights were switched off in accordance with routine practice. One scrub nurse was positioned at the operating table, assisted by a circulating nurse. The senior physician took place at the robotic console, while the second surgeon was scrubbed at the patients side. Following the start of the procedure, the anesthetia nurse left the room to simulate routine clinical flow. During the initial 4–5 min nothing besides self-limiting extrasystoles occured. Then the monitor showed ventricular fibrillation, marking the onset of cardiac arrest.

### Data collection and statistics

Following key data were recorded: primary endpoint: time from start of emergency to the beginning of CPR; secondary enpoint: time to undock. Further times were time between emergency start and beginning of undocking, time between end of undocking and beginning of CPR, time until defibrillation. The simulations were supervised by certified anesthesiologists who regularly conduct CPR simulations at the hospital. The entire simulation was recorded with a camera (Samsung Galaxy S25 Ultra, Suwon, South Corea, on a tripod). The simulation stopped upon return of spontaneous circulation (ROSC).

**Fig. 2:**
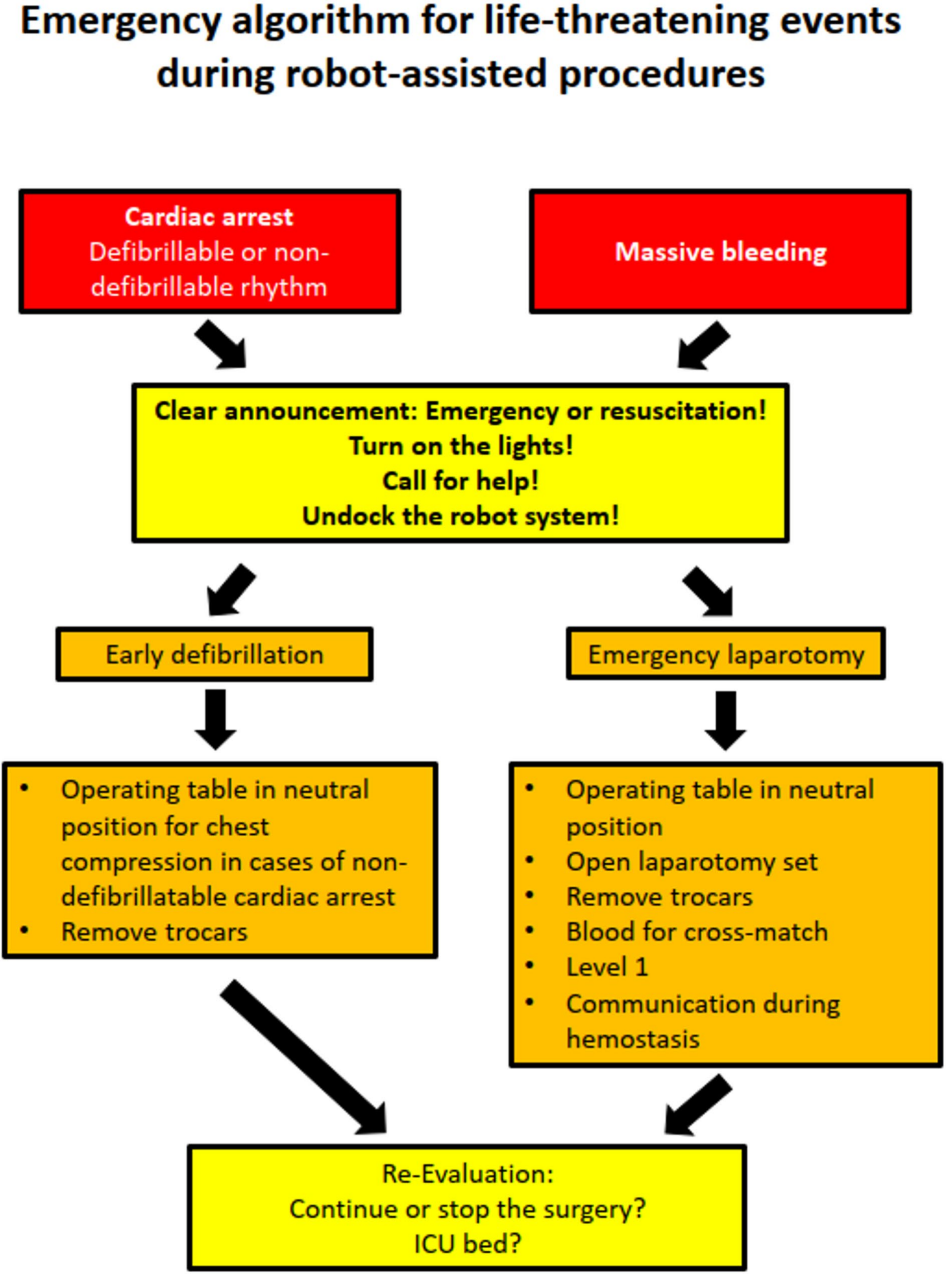
Flowchart for the procedure in an emergency situation during a robotic procedure

The scenario was conducted over three rounds, with teams reconfigured for round three. The combination of those present could vary, but each participant had completed the same number of rounds up to that point. In round 1, participants were only informed about the resuscitation simulation upon entering the operating theatre. After the first simulation a debriefing with introduction of a standardized emergency Flowchart was performed before a second simulation for the same group started at the same day (Fig. 2). Again, a debriefing was conducted. After 3–5 months a third unannounced simulation with same protocol was performed. The debriefings were conducted in the recommended structured format [17].

We recorded the time intervals between the start of the emergency and the inititation of chest compression plus defibrillation. Time intervals were measured in whole seconds using video-based analysis. We created a flowchart score based on how many of the five items in the flowchart were implemented reflecting protocol adherence as a secondary endpoint (communicate the emergency (mandatory call for help outside the OR), turn on the lights, call for help, undock (compete removal of the robotic system from the patient), reposition the table). Only the execution was evaluated, not the order. This information was collected by the training anesthesiologist present, who monitored the simulation. The data was then verified during the video reviews. The video reviews were conducted separately by a urologist and an anesthesiologist.

Data were analyzed using the Kruskal-Wallis-Test, Mann-Whitney-U-Test and the t-test. Continuous variables were reported as the mean±standard deviation, median (minimum – maximum). A p-value ≤ 0.05 was considered to indicate statistical significance. Calculations were performed with “IBM SPSS Statistics 28” (Armonk, New York, USA). The study was approved by the local ethics committee (BO-EK-73022024).

## Results

### Total times of both systems

**Fig. 3:**
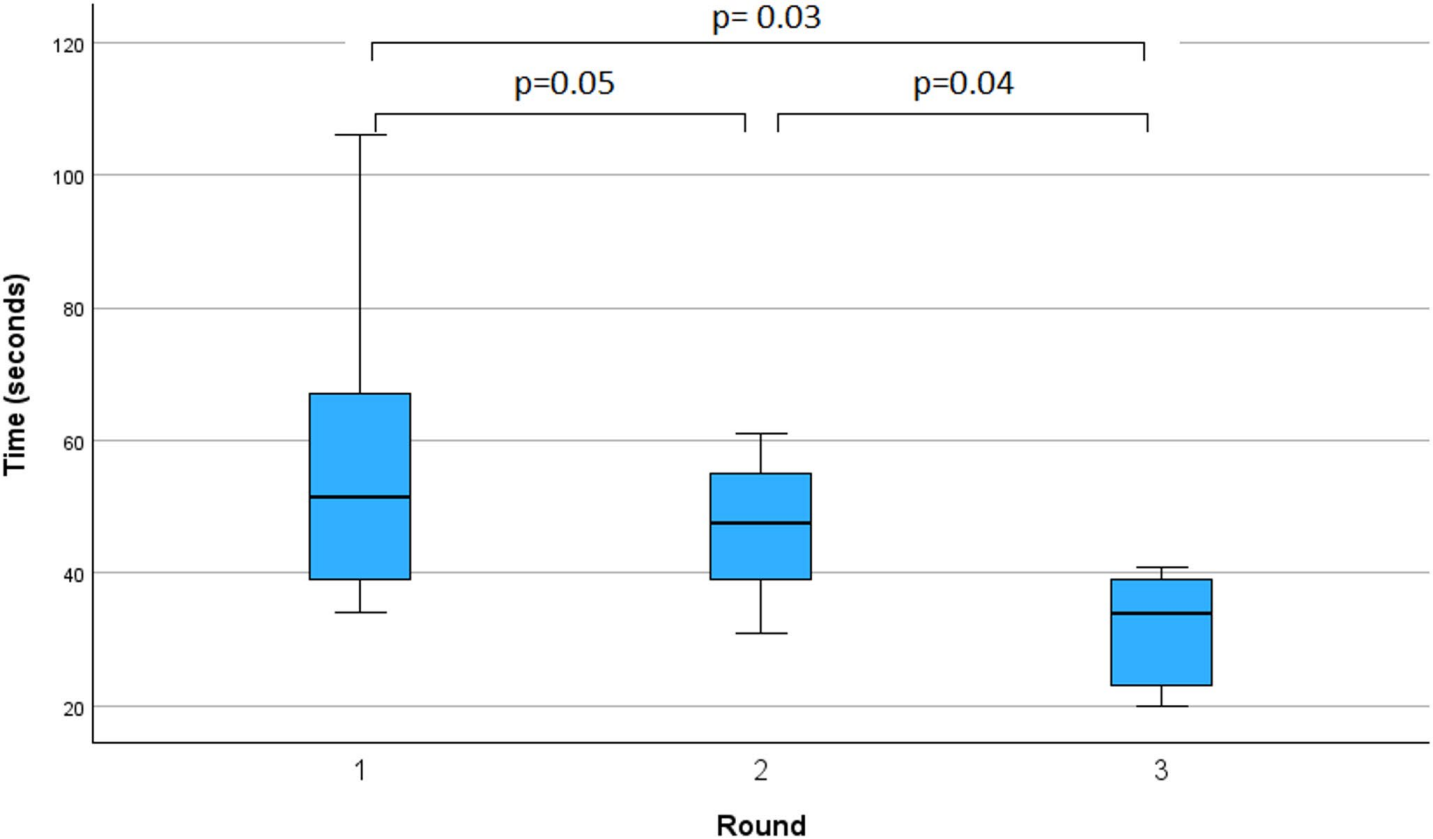
Time from the start of the emergency to the start of CPR in seconds over three rounds with six teams and daVinic^®^ & HugoRAS^®^ (*n* = 18)

There was a significant improvement of six teams in the primary endpoint: time from start of emergency to CPR initiation over the course of the three rounds (first run: 58.2 ± 26.2 s, second run: 46.8 ± 11.5 s, third round: 31.8 ± 8.9 s, *p* = 0.04). There was also an improvement in the secondary endpoint: protocol adherence as represented by the flowchart score (first run: 3.3 ± 1.0 s, second run: 4.8 ± 0.4 s, third round: 4.5 ± 0.8 s, *p* = 0.03) (Table l). Comparing the first round to the third rounds, significant improvements were observed both for the time to initiate undocking (18.5 ± 11.9 vs. 7.2 ± 4.5 s, *p* = 0.02) and for the total time to CPR (58.2 ± 26.2 vs. 31.8 ± 8.9 s, *p* = 0.03) (Fig. 3). Analysis of flow chart adherence revealed improvements in key non-technical aspects from the first to the third simulation round, including emergency communication (33% vs. 83%) and switching on the operating room lights (33% vs. 100%) (Suppl. Tbl. 3).

**Table 1:** Time periods during the emergency per round (*n* = 18, Kruskal-Wallis-test) with six teams and daVinic^®^ & HugoRAS^®^, *5 with negative times (2 courses in round 1 and 3 in round 3 with HugoRAS^®^)

Time to chest compression (seconds)	All (*n* = 18)	First round (*n* = 6)	Second round (*n* = 6)	Third round (*n* = 6)	*p* value
	45.6 ± 19.7 40 (20–106)	58.2 ± 26.2 51.5 (34–106)	46.8 ± 11.5 47.5 (31–61)	31.8 ± 8.9 34 (20–41)	0.04
Time to start undocking (seconds)	12.8 ± 9.1 12 (1–41)	18.5 ± 11.9 15 (6–41)	12.8 ± 6.3 11 (6–23)	7.2 ± 4.5 7.5 (1–12)	0.57
Undocking time (seconds)	30.9 ± 11.7 28 (18–51)	29.7 ± 12.1 25 (20–51)	30.0 ± 11.0 30 (18–46)	33.0 ± 13.9 32.5 (19–51)	0.9
Time between end of undocking until chest compression (seconds) (*n* = 13)*	9.3 ± 11.9 6 (0–44)	19.8 ± 17.9 16.5 (2–44)	4.0 ± 2.9 5 (0–7)	6.0 ± 5.2 3 (3–12)	0.3
Time to defibrillation (seconds)	88.8 ± 37.8 80 (41–186)	102.2 ± 40.9 86 (64–157)	91.3 ± 50.0 82 (41–186)	72.8 ± 12.4 67.5 (63–91)	0.4
Flowchart score	4.2 ± 1.0 5 (2–5)	3.3 ± 1.0 3 (2–5)	4.8 ± 0.4 5 (4–5)	4.5 ± 0.8 5 (3–5)	**0.03**

### Comparison of robotic systems

No significant difference was observed in overall time to initiate CPR between the daVinci^®^ and HugoRAS^®^ systems (48.0 ± 23.5 s vs. 43.2 ± 16.1 s, *p* = 0.6). However, undocking time was significantly shorter with the daVinci^®^ system (20.8 ± 2.4 vs. 41.0 ± 7.5 s, *p* < 0.001). Consequently, there was also a significant difference in the percentage of undocking during the initiation of resuscitation (*p* = 0.04). In daVinci^®^ the undocking phase accounted for 48.9 ± 14.0% of the time leading up to the CPR. In Hugo it accounted for 69.8 ± 13.1% (Table 2). Separate evaluation of rounds one through three for both systems revealed a significant improvement in the flowchart score only in the daVinci^®^ group (2.7 ± 0.6 to 4.7 ± 0.6, *p* = 0.04) (Suppl. Tbl. 1 & 2).

**Table 2:** Time periods during the emergency according robotic system with six teams(*n* = 18, t-test), *5 with negative times (2 courses in round 1 and 3 in round 3 with HugoRAS^®^)

Time to chest compression (seconds)	All (*n* = 18)	daVinci^®^ (*n* = 9)	HugoRAS^®^ (*n* = 9)	*p* value
	45.6 ± 19.7 40 (20–106)	48.0 ± 23.5 39 (30–106)	43.2 ± 16.1 41 (20–67)	0.6
Time to start undocking (seconds)	12.8 ± 9.1 12 (1–41)	15.9 ± 10.6 13 (7–41)	9.8 ± 6.4 9 (1–20)	0.158
Undocking time (seconds)	30.9 ± 11.7 28 (18–51)	20.8 ± 2.4 20 (18–26)	41.0 ± 7.5 42 (30–51)	**< 0.001**
Time between end of undocking until chest compression (seconds) (*n* = 13)*	9.3 ± 11.9 6 (0–44)	11.3 ± 13.6 6 (2–44)	4.7 ± 5.5 3.5 (0–12)	0.4
Time to defibrillation (seconds)	88.8 ± 37.8 80 (41–186)	75.9 ± 30.7 64 (41–150)	101.7 ± 41.5 91 (63–186)	0.2
Proportion of undocking in the time until the start of chest compressions (%) (*n* = 13)*	55.3 ± 16.6 53.9 (20–83)	48.9 ± 14.0 48.7 (20–67)	69.8 ± 13.1 72.0 (52–83)	**0.04**
Flowchart score	4.2 ± 1.0 5 (2–5)	4.0 ± 1.1 4 (2–5)	4.4 ± 0.9 5 (3–5)	0.4

### Additional observations

**Fig. 4:**
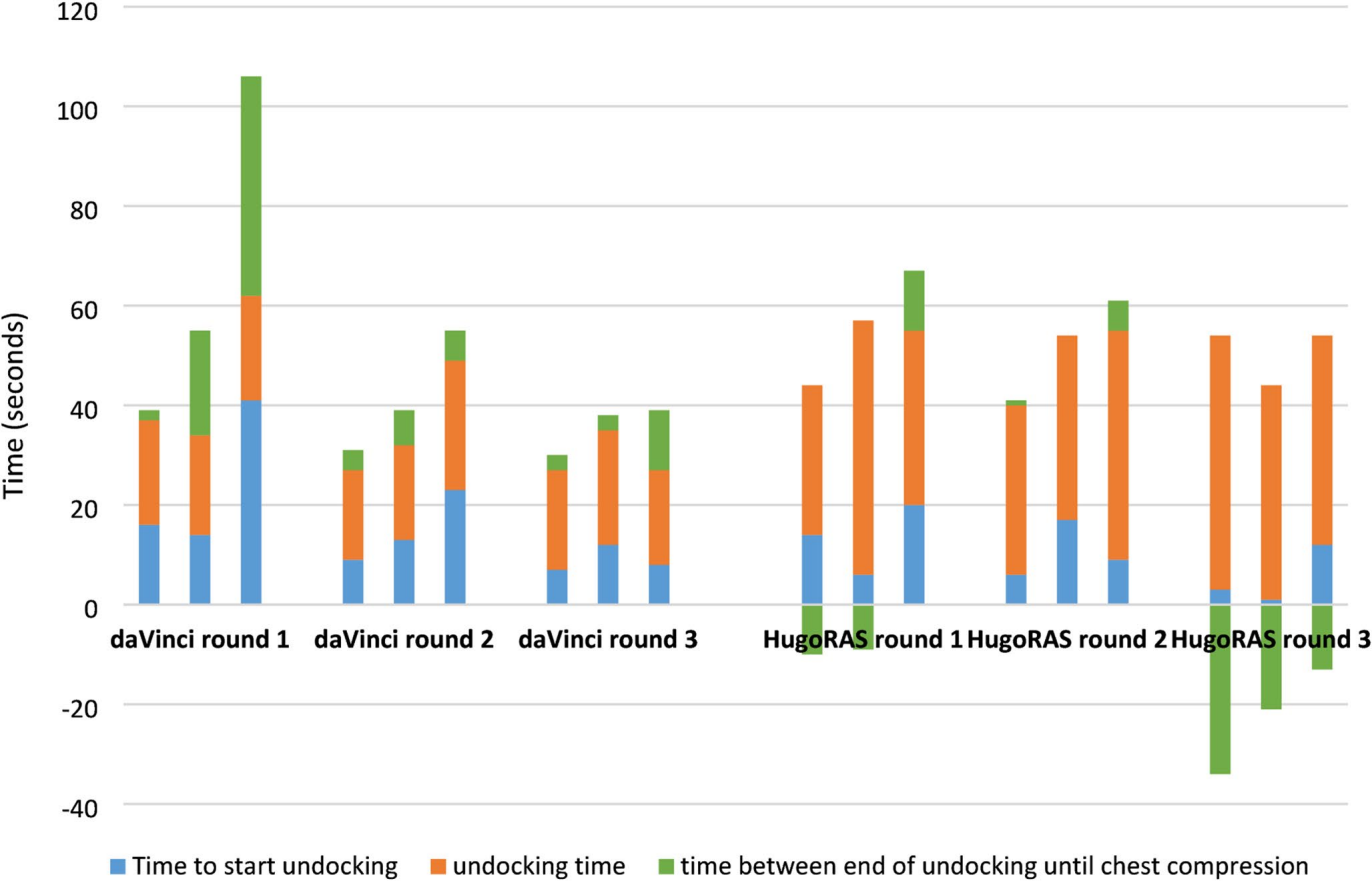
Distribution of time intervals from all 18 simulations until the start of chest compressions, broken down by robotic system with six teams and daVinic^®^ & HugoRAS^®^ (*n* = 18). Negative green bars in Hugo RAS round 3 result from the start of resuscitation before the completion of undocking

Two unexpected observations were made during resuscitation: First, in 6 out of 9 cases (67%), acoustic warning signals were triggered when the HugoRAS^®^ system was undocked due to contact with the contact strips, resulting in increased ambient noise in the operating theatre, potentially impairing communication during resuscitation. Second, in 5 out of 18 cases, chest compressions were started before the undocking process was completed. All 5 cases were performed with the HugoRAS^®^ system. In 2 cases this was done in the first run. In 3 cases, this was done in the third run (Fig. 4). The time interval during which chest compressions were administered during undocking ranged from 9 to 34 s. In all 5 cases, the instruments had already been removed and a maximum of two arms were still docked to trocars.

## Discussion

Through simulation-based training, we demonstrate a learning effect with a significant reduction in the time taken to begin CPR over three rounds (58.2 ± 26.2 vs. 31.8 ± 8.9 s, *p* = 0.03). Compliance with the steps in the flowchart also improved significantly from round 1 to round 3. Furthermore, a comparison of the robotic systems shows a significantly shorter undocking time for the daVinci^®^ system (20.8 ± 2.4 vs. 41.0 ± 7.5 s, *p* < 0.001), although this has no statistical influence on the time to CPR.

Resuscitation during robotic surgery remains underrepresented in the literature despite the increasing number of robotic procedures performed worldwide. While several case reports describe intraoperative emergencies managed without undocking or CPR [11–13, 15], only two reports detailed CPR during ongoing robotic surgery. In these cases, anesthesiologic emergency management is described retrospectively, as documentation typically focuses on therapeutic interventions rather than the precise timeline from onset of cardiac arrest to initiation of CPR—data that are rarely captured during real emergencies [14, 18]. Although reliable data on the incidence of CPR during robotic surgery are lacking, the clinical relevance of this scenario is widely acknowledged. Several groups have developed emergency algorithms and evaluated them in simulation-based training. Melnyk et al. has shown that simulation training improves confidence, knowledge, and skills [19]. Huser et al. were able to show that training reduced the time required for CPR, removal of the robotic system, defibrillation and ROSC [20]. Similiary Patki et al. implemented an emergency flowchart and reported improvements in teamwork scores as well as shorter times to CPR and undocking [21].

Our findings are consistent with these studies. We demontrated reduced time to CPR from 58 to 31 s, compared with reductions from 101 to 48 s reported by Patki and from 71 to 25 s reported by Huser. Patki showed a reduction in undocking time from 86 to 25 s while Huser reported a decrease from approximately 110 to 40 s [20, 21]. In our study, undocking time with the daVinci^®^ system decreased from 23.7 to 9.0 s (not statistically significant), whereas no meaningful reduction was observed for HugoRAS^®^ (52.0 to 50.0 s). The comparatively short initial undocking times for daVinci^®^ likely reflect a high level of staff experience. Unlike previous studies, we conducted a third unannounced simulation 3–5 months later, demonstrating an ongoing progress in training.

Direct comparison of the two robotic systems showed no significant difference in time to CPR (48.0 ± 23.5 vs. 43.2 ± 16.1 s, *p* = 0.6). This finding is unexpected given the significantly shorter undocking time of the daVinci^®^ system. Numerous studies have compared docking times between HugoRAS^®^ and daVinci^®^, generally showing longer docking times for HugoRAS^®^ [22–25], . Although this can be reduced with experience, but it still cannot match the daVinci system [24, 26].

In a previous study addressing docking time between these robotic platforms conducted by our group, undocking required 5.6 min for HugoRAS^®^ and 4.0 min for daVinci^®^ [25]. Notably, the study personnel were identical to the participants included in the present investigation. In the emergency simulations, undocking times were markedly reduced: from 4.0 min to 20.8 s for daVinci^®^ (approximately sixfold faster) and from 5.6 min to 41.0 s for HugoRAS^®^ (approximately eightfold faster). These shorter times reflect, on the one hand, the fact that the undocking procedures in the cited study were performed on actual patients and, on the other hand, that in the current study there was time pressure to remove the arms even more quickly due to the emergency. Nevertheless, the time differences illustrate just how much the undocking process can be accelerated in an emergency. Given that undocking accounts for a substantial portion of the interval before initiation of chest compressions (Fig. 4), the absence of a system-related difference in CPR initiation warrants further consideration.

Several factors may explain this observation. First, the limited number of simulations per system increases susceptibility to statistical variability. In one daVinci^®^ simulation, a considerable delay occurred between completion of undocking and initiation of CPR (Fig. 4). Second, video analysis revealed that in 5 of 9 HugoRAS^®^ simulations, chest compressions were initiated before full undocking was completed. Workflow differences may also contribute: in the daVinci^®^ system, the bedside assistant removes the camera arm and right instrument arm while sterile nurses remove the two left arms. In contrast, with HugoRAS^®^, the sterile nurse removes the camera and two left-sided arms (all positioned on the patient’s left), leaving the bedside assistant responsible only for the right instrument arm—thereby allowing earlier initiation of compressions. Importantly, in all simulations, all instruments were fully removed once chest compressions had begun.

These findings raise the question of whether CPR may be safely initiated once instruments are removed, even if robotic arms are not yet completely cleared. Considering the recommendation to begin CPR within 2 min, this threshold was consistently met [10]. In this regard, the definition should be that CPR should begin once the robot is completely undocked. Including nuances in the protocol—such as allowing CPR to begin once the instruments have been removed even though the arms are still docked—could also carry the risk that CPR is initiated while instruments are still inside the body. Furthermore, a fully undocked robot is easier to verify than determining whether an instrument is still inside or not. Another unexpected finding was the loud error messages emitted by the Hugo robot. These did not appear to have any effect on the resuscitation process. However, the question remains as to whether they exacerbate the stressful situation for the staff present. Ultimately, it must be concluded that resuscitation protocols should, in principle, be universally applicable to robotic systems. The first simulation round—particularly one outlier with a time of 106 s—illustrates the risk of delayed CPR initiation in inexperienced teams (daVinci round 1 in Fig. 4). This underscores the importance of structured algorithms and regular team training.

This study has several limitations. First, the sample size was small with six teams. A larger sample size would have yielded statistically more robust results. Nevertheless, six teams represent a total of 12 surgical nurses experienced in robotic systems—which is a significant number. A larger sample size would be possible almost exclusively in multicenter studies. Second, it was conducted in a simulated environment, which may not fully reflect the complexity, stress, and time pressure of real intraoperative cardiac arrest. Consequently, the transferability of the observed improvements to clinical practice may be limited. Third, the study was performed at a single high-volume center with a limited number of teams, restricting statistical power and generalizability. As team composition was not constant across simulation rounds, this may have impacted overall performance outcomes; however, this approach was chosen to reflect routine clinical practice. Fourth, improvements observed across simulation rounds may partly reflect a repetition effect and clinical experience gained in the interim between Round 2 and Round 3 in addition to the structured debriefing and introduction of the emergency algorithm. Finally, surrogate endpoints such as time to initiation of chest compressions were used, as clinical outcome data are not feasible for such rare intraoperative events. Fifth, the emergency scenario focuses on emergencies involving vital signs and does not address surgical emergencies such as severe bleeding or the concept of conversion. Accordingly, in our scenario, the anesthesiologist was in charge of emergency management. In the case of surgical emergencies, this responsibility would fall to the surgeon.

Despite these limitations, this study represents one of the few investigations specifically addressing CPR during robotic surgery and highlights the critical importance of time-focused training. To our knowledge, it is also the first study to include the HugoRAS^®^ system. Although concerns remain regarding CPR timing with HugoRAS^®^—given longer docking times but earlier initiation of compressions prior to complete undocking—the observed time intervals remained within acceptable limits and are unlikely to adversely affect patient survival compared with daVinci^®^. Future multicenter studies are warranted to validate these findings and to evaluate their impact on patient-centered outcomes. In addition, other positions should also be practiced—for example, the lateral position during kidney surgery. Nevertheless, the robot-assisted CPR training will be incorporated into the CPR training program at the local hospital, which all staff members are required to complete once a year.

## Conclusions

Despite the presumed low incidence of cardiopulmonary resuscitation during robotic procedures and the scarcity of published data, the technical complexity of robotic systems—particularly the need for coordinated undocking and effective communication with the console surgeon—demands special consideration. Simulation-based multidisciplinary team training may significantly improve resuscitation performance during robotic-assisted surgery and leads to faster initiation of CPR. Undocking constitutes the principal source of delay, with relevant system-specific differences, although these do not necessarily translate into prolonged time to CPR.

## Electronic Supplementary Material

Below is the link to the electronic supplementary material.

Supplementary Material 1

## Data Availability

No datasets were generated or analysed during the current study.
