# Supplementary material for: Resuscitation during robotic-assisted pelvic surgery: impact of simulation training and system-specific factors
Source: J Robot Surg. 2026 Apr 13;20(1):434. doi: 10.1007/s11701-026-03399-1 (PMC13070981; doi:10.1007/s11701-026-03399-1)
Supplement: Supplementary file 1 — Supplementary Material 1 [file 11701_2026_3399_MOESM1_ESM.docx]

**Suppl. Table 1**: Time periods during the emergency according for round 1 and 3 with daVinci® system with 3 teams (n=6, Mann-Whitney-U-test).

|  | **All daVinci**  **(n=6)** | **First round**  **(n=3)** | **Third round**  **(n=3)** | **p value** |
| --- | --- | --- | --- | --- |
| Time to chest compression (seconds) | 51.2±28.7  39 (30 – 106) | 66.7±35.0  55 (39 – 106) | 35.7±4.9  38 (30 – 39) | 0.08 |
| Time to start undocking (seconds) | 16.3±12.6  13 (7 – 41) | 23.7±15.0  16 (14 – 41) | 9.0±2.6  8 (7 – 12) | 0.05 |
| Undocking time (seconds) | 20.7±1.4  20.5 (19 – 23) | 20.7±0.6  21 (20 – 21) | 20.7±2.1  20 (19 – 23) | 0.7 |
| Time to end undocking (seconds) | 37.0±12.9  34.5 (27 – 62) | 44.3±15.4  37 (34 – 62) | 29.7±4.6  27 (27 – 35) | 0.1 |
| Time between end of undocking until chest compression (seconds) | 14.2±16.4  7.5 (2 – 44) | 22.3±21.0  21 (2 – 44) | 6.0±5.2  3 (3 – 12) | 0.5 |
| Time to defibrillation (seconds) | 51.2±28.1  39 (30 – 106) | 66.7±35.0  55 (39 – 106) | 35.7±4.9  38 (30 – 39) | 0.3 |
| Flowchart score | 3.7±1.2  3.5 (2 – 5) | 2.7±0.6  3 (2 – 3) | 4.7±0.6  5 (4 – 5) | **0.04** |

**Suppl. Table 2**: Time periods during the emergency according for round 1 and 3 with HugoRAS® system with 3 teams (n=6, Mann-Whitney-U-test), *5 with negative times (2 courses in round 1 and 3 in round 3 with HugoRAS®).

|  | **All Hugo**  **(n=6)** | **First round**  **(n=3)** | **Third round**  **(n=3)** | **p value** |
| --- | --- | --- | --- | --- |
| Time to chest compression (seconds) | 38.8±17.4  37.5 (20 – 67) | 49.7±16.6  48 (34 – 67) | 28.0±11.4  23 (20 – 41) | 0.1 |
| Time to start undocking (seconds) | 9.3±7.3  9 (1 – 20) | 13.3±7.0  14 (6 – 20) | 5.3±5.9  3 (1 – 12) | 0.1 |
| Undocking time (seconds) | 42.0±8.4  42.5 (30 – 51) | 38.7±11.0  35 (30 – 51) | 45.3±4.9  43 (42 – 51) | 0.4 |
| Time to end undocking (seconds) | 51.3±5.8  54 (44 – 57) | 52.0±7.0  55 (44 – 57) | 50.7±5.8  54 (44 – 54) | 0.4 |
| Time between end of undocking until chest compression (seconds) (n=1)* | 12 | 12 | - | * |
| Time to defibrillation (seconds) | 92.5±33.5  85.5 (63 – 157) | 109.7±41.4  92 (80 – 157) | 75.3±14.3  72 (63 – 91) | 0.1 |
| Flowchart score | 4.2±1.0  4.5 (3 – 5) | 4.0±1.0  4 (3 – 5) | 4.3±1.2  5 (3 – 5) | 0.6 |

**Suppl. Table 3**: Flowchart items during the emergency per round with six teams and daVinic® & HugoRAS®.

| **Flowchart items** | **All (n=18)** | **First round (n=6)** | **Second round (n=6)** | **Third round (n=6)** |
| --- | --- | --- | --- | --- |
| Emergency communication | 13 (72%) | 2 (33%) | 6 (100%) | 5 (83%) |
| Switching on the lights | 13 (72%) | 2 (33%) | 5 (83%) | 6 (100%) |
| Call for help | 15 (83%) | 5 (83%) | 6 (100%) | 4 (67%) |
| Undocking | 18 (100%) | 6 (100%) | 6 (100%) | 6 (100%) |
| Repositioning of the operating table | 17 (94%) | 5 (83%) | 6 (100%) | 6 (100%) |
